# Effects of the seedling tray overlapping for seed emergence mode on emergence characteristics and growth of rice seedlings

**DOI:** 10.3389/fpls.2024.1341318

**Published:** 2024-03-15

**Authors:** Yizhuo Gao, Meihong Shao, Yuping Zhang, Yikai Zhang, Yaliang Wang, Zhigang Wang, Defeng Zhu, Yunbo Zhang, Jing Xiang, Huizhe Chen

**Affiliations:** ^1^ China National Rice Research Institute/State Key Laboratory of Rice Biology, Hangzhou, China; ^2^ MARA Key Laboratory of Sustainable Crop Production in the Middle Reaches of the Yangtze River/College of Agriculture, Yangtze University, Jingzhou, China; ^3^ Jiande Agricultural Technique Extension Center, Hangzhou, China

**Keywords:** rice, seedling raising mode, seed plumpness, seedling emergence characteristics, seedling quality, nutrient uptake

## Abstract

Seedling mode plays a crucial role in the rice production process, as it significantly affects the growth and development of seedlings. Among the various seedling modes, the seedling tray overlapping for seed emergence mode (STOSE mode) has been demonstrated to be effective in enhancing seedling quality. However, the impact of this mode on the germination and growth of seeds with varying plumpness remains uncertain. To investigate the effect of the STOSE mode on seedling emergence characteristics, growth uniformity, and nutrient uptake of seeds with varying plumpness levels, we conducted a study using super early rice Zhongzao 39 (ZZ39) as the test material. The seeds were categorized into three groups: plumped, mixed, and unplumped. The results indicated that the STOSE mode significantly improved the seedling rate for all types of seeds in comparison to the seedling tray nonoverlapping for seed emergence mode (TSR mode). Notably, the unplumped seeds exhibited the most pronounced enhancement effect. The soluble sugar content of the seeds increased significantly after 2 days of sowing under the STOSE mode, whereas the starch content exhibited a significant decrease. Furthermore, the STOSE mode outperformed the TSR mode in several aspects including seedling growth uniformity, aboveground dry matter mass, root traits, and nutrient uptake. Overall, the STOSE mode not only promoted the germination and growth of plumped and mixed seeds but also had a more pronounced impact on unplumped seeds.

## 1 Introduction

Rice (*Oryza sativa* L.) is a vital food crop, with approximately 50% of the world’s population relying on it as a primary source of sustenance (Tian et al., 2022). Furthermore, with the global population steadily increasing, the demand for rice continues to rise annually (Muthayya et al., 2014). China is a major producer and consumer of rice in the world. However, the traditional manual cultivation methods are inadequate for the current development needs, due to the rapid economic growth and urbanization in recent years that have caused a large transfer of the rural labor force (Chen et al., 2019). Mechanized rice production significantly decreases the reliance on manual labor and contributes greatly to enhancing labor productivity and increasing yields and profits. It represents the developmental trajectory of modern rice farming technology (Palacios-Lopez et al., 2017; Emran and Shilpi, 2018; Mukhamedova and Wegerich, 2018; Zhu et al., 2019). Furthermore, mechanized rice planting relies on machine transplantation. The core of machine transplantation is cultivating seedlings (Yu et al., 2006). The traditional method of raising seedlings is highly susceptible to environmental influences. This often leads to issues including low seedling emergence rates, uneven emergence, and poor quality seedlings. Consequently, machine-transplanted seedlings frequently have high leakage rates, poor uniformity, and reduced quality. Ultimately, these factors restrict improvements in rice yield as well as the development and application of machine transplanting technologies (Zheng et al., 2014). To address the aforementioned problems with rice planting, the China National Rice Research Institute (CNRRI) adopted the seedling tray overlapping for seed emergence mode (STOSE mode). It also developed the necessary facilities, equipment, and seedling cultivation techniques to support this mode. The mode uniformly employs stackable trays to sow seeds. The trays are then transferred to the seedling center which provides suitable temperature and humidity conditions for germination. Once the seedlings reach 0.5 cm in length, the trays are shipped to seedling farms for further cultivation. The farm includes greenhouses, dry paddy field nurseries, and so on. Consequently, a “1+N” mode has been established, whereby one seedling center raises seedlings for multiple seedling farms (Zhu et al., 2018). This mode centralizes seedling cultivation and supply, broadening the scale of seedling availability and improving service capacity. Centralized seedling supply reduces the risks and costs of production while enabling larger-scale, specialized, and intensified rice seedling cultivation.

Rice seed germination, a critical stage of the life cycle, directly impacts seedling growth and development (Bewley, 1997; Donohue et al., 2010). The germination rate and speed depend on the temperature and soil moisture content (Kebreab and Murdoch, 2000). Previous studies have shown that high temperatures inhibit seed germination in model plants such as Arabidopsis and crops such as sunflower and rice (Chiu et al., 2012; Wen, 2015; Mangrauthia et al., 2016). Moreover, low temperatures decrease gibberellin (GA) and increase abscisic acid (ABA) levels in rice seeds. This alters hormone balances, slowing seed development. A study by Wang et al. (2018) also demonstrated that low temperatures reduce the biological activity of gibberellins, impairing soluble sugar utilization in rice seeds. This effect is closely associated with impaired seed germination and seedling formation, as soluble sugars are critical energy sources for these processes. Furthermore, water availability is also critical for inducing seed germination (Bradford, 1990). Previous studies have demonstrated that drought increases abscisic acid (ABA) and reactive oxygen species (ROS) levels in rice seeds, both of which inhibit germination (Liu et al., 2019). Therefore, providing optimal temperature and humidity conditions tailored to different species will promote seed germination and seedling formation. However, traditional seedling methods use seedling trays that are sown and then flattened. These methods cause unstable temperature and humidity for germination and seedling production, leading to poor seedling quality and affecting machine transplanting. STOSE mode uses customized stackable trays that provide heat preservation and moisture retention. This mode can maintain soil moisture stability and reduce water loss, according to Wang et al. (2022b). The seedling center also uses temperature and humidity control equipment to provide suitable conditions for seed germination. There are variations in seed development at different grain positions on the rice spike. Generally, grains situated on the upper panicle primary branches exhibit earlier flowering, faster irrigation, and higher seed plumpness compared to grains on the lower secondary branches (Yang et al., 2006). It has been reported that the plumped seeds exhibited a significantly higher germination rate and seed vigor compared to unplumped seeds (Zheng et al., 2022).

Many studies have confirmed that the STOSE mode has a high seedling rate, seedlings uniformity, and better seedling quality (Shou et al., 2017; Chen et al., 2020; Wang et al., 2022b). However, the effects of the STOSE mode on the germination and seedling quality of variable rice seed plumpness have rarely been reported. In this study, we used super early rice Zhongzao 39 (ZZ39) as a test material, categorizing seeds as plumped, mixed, and unplumped by screening. Seedling emergence traits and quality among the different seed plumpness were compared under STOSE mode, with the seedling tray nonoverlapping for seed emergence mode (TSR mode) as a control. The objective of this study is to establish a theoretical foundation and offer practical recommendations for the innovation and enhancement of rice seedling modes.

## 2 Materials and methods

### 2.1 Experimental materials and environments

**Figure 1:**
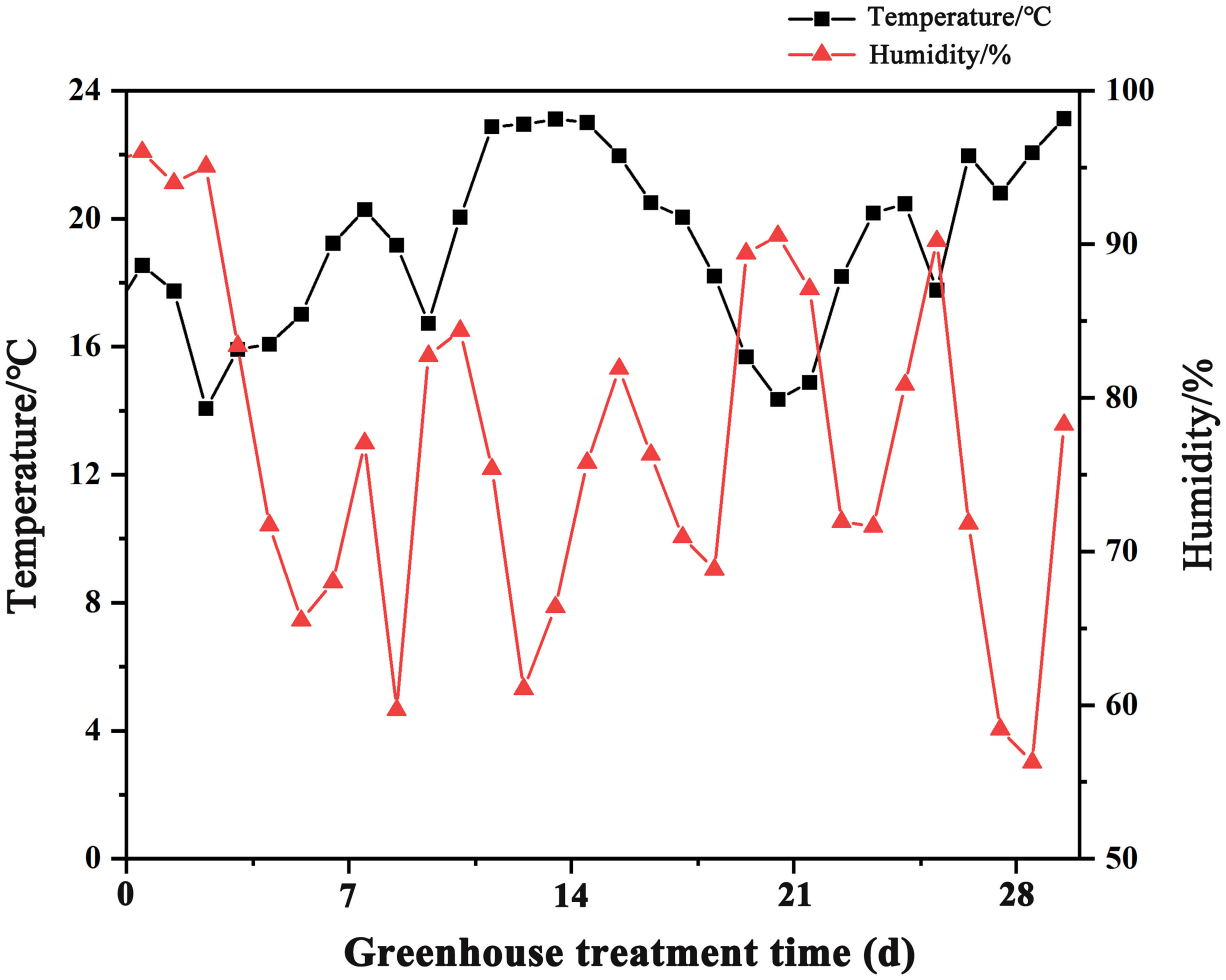
Changes of temperature and humidity in greenhouses.

This experiment was conducted in 2023 at the China National Rice Research Institute. We used Zhongzao 39 (ZZ39), a popular and widely cultivated rice variety, as the test material. Empty grains were removed by a rice wind sorter, followed by screening of unplumped seeds using 15% NaCl solution. The seedling tray overlapping for seed emergence mode (STOSE mode) was simulated by us in a thermostatic incubator with a temperature of 30°C and a humidity of 90%. We performed the seedling tray nonoverlapping for seed emergence mode (TSR mode) in a greenhouse and recorded temperature and humidity changes for one month after sowing, as illustrated in **Figure 1**.

### 2.2 Experimental design

The experiment compared two seedling cultivation modes: the STOSE and TSR modes. Three seed types were tested under each mode: plumped seeds (T1; 1000-grain weight 27.05 g), mixed seeds (T2, 80% plumped and 20% unplumped seeds), and unplumped seeds (T3, 1000-grain weight 16.88 g). The experiment consisted of six treatments, with each treatment sown in six seedling trays. Stackable seedling trays (30 cm x 25 cm) were used for the STOSE mode, while conventional flat trays (60 cm x 30 cm) were used for the TSR mode. The same rice-specific substrate was used uniformly as the seedling soil in both cultivation modes. Before sowing, the seeds were soaked for 48 hours and sterilized using a fungicide.

The amount of seed sown varied between treatments. The number of plumped seeds (T1) needed to sow 80 g of dry grain per tray under the TSR mode served as the reference to calculate sowing quantities for the mixed (T2) and unplumped seed treatments (T3). The goal was to use the same number of seeds per tray under the TSR mode. Sowing quantities for the plumped, mixed and unplumped seed treatments under the STOSE mode were calculated to match the sowing density used under the TSR mode. Under the TSR mode, trays are laid flat in greenhouses after sowing and managed as dry nursery seedlings. Under the STOSE mode, trays were placed in a constant temperature incubator for 36 h and then moved to greenhouses, where they received the same treatment as the TSR mode. Seedling samples were collected at 2, 6, 10, 18, and 30 days after sowing to examine the effects of different treatments on seedling quality (plant height, aboveground and root dry weight), growth uniformity, root traits (total root length, root surface area, root volume and number of roots), seed sugar and starch content, and aboveground nutrient uptake.

### 2.3 Measurements and methods

Prior to the experiment, 50 seeds of each seed type (plumped, mixed and unplumped) were sown into 7 cm diameter circular positioning rings (made of hollow nylon tubing). Three seedling trays per treatment were chosen, with three positioning rings placed evenly in each tray. The number of seedlings from each treatment was investigated, starting 2 days after sowing when the emergence length was approximately 5 mm. Subsequent surveys were conducted every 2 days until 10 days after sowing. The seedling rate was calculated using Equation 1 (Wang et al., 2022a):


(1) $$\begin{array}{c} \text{seedling rate }\left(\%\right)=\text{ number of seedlings}/\\ \left(\text{number of seedlings}+\text{number of non}-\text{seedlings}\right)\times 100\%. \end{array}$$

The study investigated the plant height and growth uniformity of seedlings in each treatment at 18 and 30 days after sowing. Each treatment had three replications, and 100 seedlings were investigated in each replication. The uniformity of seedling growth was calculated using the following formula:


(2) $$\begin{array}{cc} Usg=\left(1-S_{sh}/{\bar{x}}_{sh}\right)\times 100\;; & S_{sh}=\sqrt{1/\left(n-1\right)\sum_{i=1}^{n}{\left(xi_{sh}-{\bar{x}}_{sh}\right)}^{2}} \end{array}$$

*S_sh_* indicates the standard deviation of seedling heights; *xi_sh_* indicates seedling height observations; ${\bar{x}}_{sh}$ indicates the average seedling height (Equation 2) (Wang et al., 2022b).

Moreover, the root systems of the seedlings were cleaned and scanned with a plant root scanner (Epson Perfection V700 Photo). The scanned images were stored in a computer and analyzed by Win-RHIZO PRO 2013, a root analysis system software. The software measured parameters such as total root length, number of roots, root surface area and root volume. The seedlings were oven-dried at 80°C until they reached a constant weight. Then, the dry weights of the aboveground and root parts were measured separately.

The anthrone method was utilized to determine soluble sugar content in seeds (Zhou et al., 2009). Seed samples from each treatment were collected at 2, 6, and 10 days after sowing, cleaned, dried, and milled. Seeds prior to sowing served as experimental controls. For each sample, 0.1 g of seeds was weighed into 10 ml reagent tubes, combined with distilled water, and homogenized. Samples underwent hot water extraction in a boiling water bath (temperature exceeding 95°C) for 30 min, followed by centrifugation at 8000 rpm for 10 min. The resulting supernatants were collected in 25 ml volumetric flasks, and the extraction procedure was repeated twice. Pooled supernatants were brought up to volume, comprising the soluble sugar extract. The absorbance values of the samples were determined using a spectrophotometer at a wavelength of 630 nm. The starch content of seeds was measured by the perchloric acid method (Wang et al., 1993). The experiment involved six treatments, with three replications for each treatment.

The nitrogen (N), phosphorus (P), and potassium (K) contents in the aboveground parts of seedlings were measured according to a previously reported method (Zuo et al., 2023). Samples were collected from different treatments at 18 and 30 days after sowing. Only the aboveground parts of the seedlings were retained and dried before being ground. A 0.1 g sample was then weighed in a test tube and decocted by adding concentrated H_2_SO_4_-H_2_O_2_. The resulting solution was fixed in a 50 ml volumetric flask, which served as the solution to be measured. The total nitrogen (N), total phosphorus (P), and total potassium (K) contents of the aboveground seedlings were measured using the Kjeldahl nitrogen determination, molybdenum-antimony colorimetric method, and flame photometric method, respectively. The N, P, and K uptake under different treatments was calculated using Equation 3 (Hu et al., 2019):


(3) N(P,K) uptake (mg)=seedling dry weight (g)×N(P,K) content (%).

### 2.4 Statistical analysis

In this study, the data were organized and calculated using Excel 2021 software, and SPSS 23.0 statistical software was used to perform three repeated one-way analyses of variance (ANOVA) and Duncan’s multiple comparisons tests. All values were reported as the means ± SD (standard deviation). Pictures were drawn using SigmaPlot 10.0 software.

## 3 Results

### 3.1 Seedling rate

**Figure 2:**
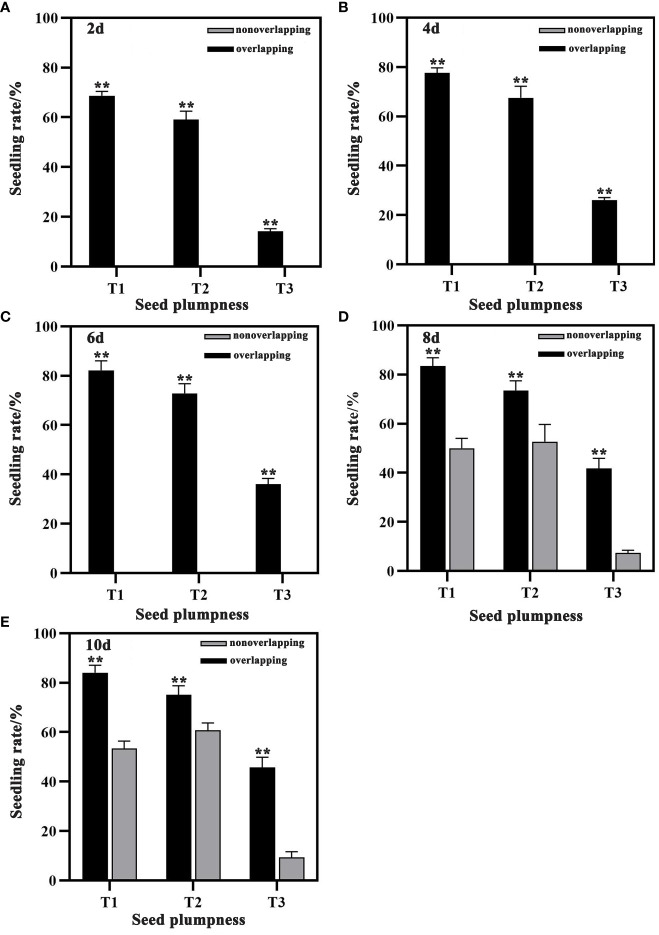
Changes in seedling rate of each seed treatment under STOSE and TSR modes. Nonoverlapping and overlapping represent TSR and STOSE modes, respectively. 2d, 4d, 6d, 8d and 10d indicate the number of days after sowing. T1, T2 and T3 represent different types of seeds (plumped, mixed and unplumped seeds, respectively). Bars mean SD (n=3). **, indicates that the difference in seedling rate of the same type of seeds under different seedling modes was highly significant (P<0.01, LSD method).

**Figure 3:**
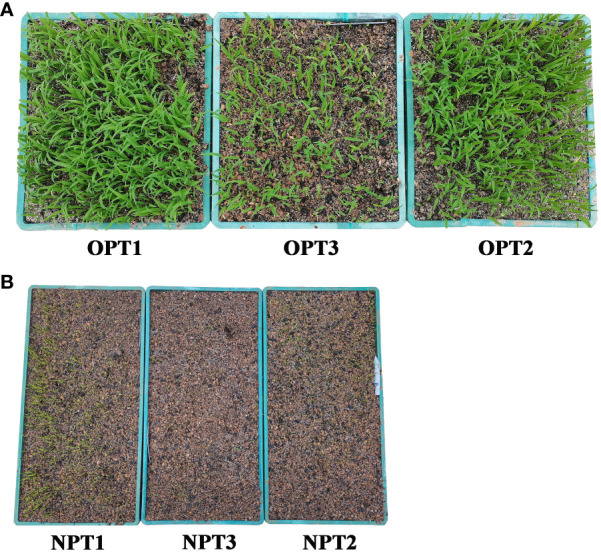
Pictures of seedling emergence under different treatments at 10d after sowing. Row **(A)** shows the seed treatments under STOSE mode: OPT1 for plumped seeds, OPT2 for mixed seeds and OPT3 for unplumped seeds. Row **(B)** shows the seed treatments under TSR mode: NPT1 for plumped seeds, NPT2 for mixed seeds and NPT3 for unplumped seeds.

Seedling rates were significantly higher under the STOSE mode than the TSR mode across all seed treatments. Under the STOSE mode, the seedling rates over time followed the trend of T1 > T2 > T3 (**Figure 2**). The seeds under the STOSE mode sprouted into seedlings at 2 days after sowing and increased gradually until 10 days, when the seedling rate stabilized (**Figure 3A**). The seedling rate of the T3 treatment under the STOSE mode showed a significant increase of 82.84 and 153.16% at 4 and 6 days after sowing, respectively, compared to 2 days (**Figures 2A–C**). Under the TSR model, the seeds sprouted into seedlings at 8 days after sowing and reached a stable seedling rate at 10 days (**Figure 3B**). The seedling rates of T1, T2 and T3 increased by 6.66, 15.19 and 27.29%, respectively, from 8 to 10 days after sowing. Compared to the TSR mode, the STOSE mode increased the seedling rates of T1, T2 and T3 by 67.11, 39.66 and 469.7%, respectively, at 8 days after sowing. The increase was lower at 10 days after sowing, with 57.50, 23.81 and 390.48% for T1, T2 and T3 (**Figures 2D, E**). The results indicate that the STOSE mode can significantly enhance the seedling rate compared to the TSR mode. The enhancement effect was more pronounced for unplumped seeds than for other seed types.

### 3.2 Soluble sugar and starch content

**Figure 4:**
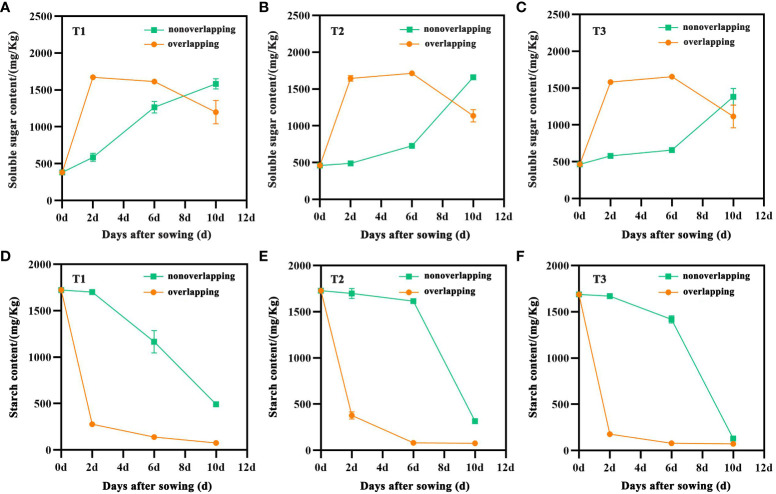
Changes in soluble sugar and starch content of seeds under different treatments within 10 d after sowing. Nonoverlapping and overlapping represent TSR and STOSE modes, respectively. T1, T2 and T3 represent different types of seeds (plumped, mixed and unplumped seeds, respectively). Bars mean SD (n=3).

The seed soluble sugar content in all treatments under the STOSE mode exhibited a variation over time, initially increasing and then decreasing. The TSR mode demonstrated an overall increasing trend. In contrast, the seed starch content decreased with time under both seedling modes (**Figure 4**). The soluble sugar content of the seeds in the T1, T2, and T3 treatments under the STOSE mode exhibited a significant increase within 2 days after sowing. T1 reached its maximum level at 2 days (**Figure 4A**), whereas T2 and T3 showed a slight further increase before declining at 6 days (**Figures 4B, C**). Within the first 6 days after sowing, the soluble sugar content of seeds was significantly lower under the TSR mode than the STOSE mode. However, at 10 days after sowing, the trend reversed with the TSR treatments showing significantly higher soluble sugar content. The changes in seed starch content were significantly different between the STOSE and TSR modes. Under the STOSE mode, the T1, T2, and T3 treatments showed a significant decrease within 2 days after sowing, followed by a slower decreasing trend. On the other hand, under the TSR mode, the T1, T2, and T3 treatments exhibited a slow decrease in seed starch content within 2 days after sowing, followed by a sharp decrease (**Figures 4D–F**).

### 3.3 Seedling height and growth uniformity

**Figure 5:**
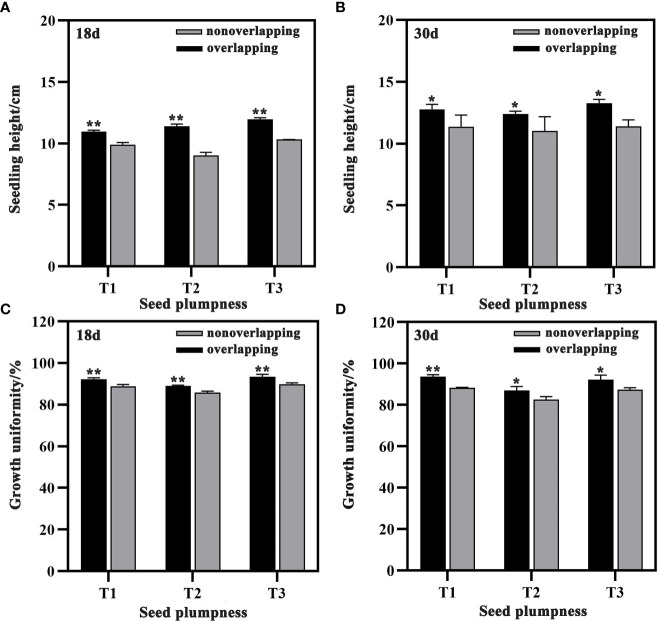
Differences in seedling height and growth uniformity of seeds with different plumpness under two seedling modes. Nonoverlapping and overlapping represent TSR and STOSE modes, respectively. 18d and 30d indicate the number of days after sowing. T1, T2 and T3 represent different types of seeds (plumped, mixed and unplumped seeds, respectively). There were three replicates for each treatment and each replicate included 100 seedlings. Bars mean SD (n=3). *, indicates that the difference in seedling height (or growth uniformity) of the same type of seeds under different seedling modes was significant (P<0.05, LSD method); **, indicates that the difference in seedling height (or growth uniformity) of the same type of seeds under different seedling modes was highly significant (P<0.01, LSD method).

Seedling height was significantly higher under the STOSE mode than the TSR mode for all treatments at both 18 and 30 days after sowing (**Figure 5**). Compared to the TSR mode, seedling height increased by 10.63, 26.33 and 15.93% for T1, T2, and T3, respectively, at 18 days under the STOSE mode (**Figure 5A**). Similarly, at 30 days, the increase was 12.40, 12.34 and 16.52% for T1, T2, and T3, respectively (**Figure 5B**). The seedling growth uniformity of each treatment was higher and significantly different under the STOSE mode than the TSR mode at both 18 and 30 days after sowing (**Figures 5C, D**). These results indicate that the STOSE mode not only enhances seedling growth but also improves the uniformity of seedling.

### 3.4 Shoot and root dry weight

**Figure 6:**
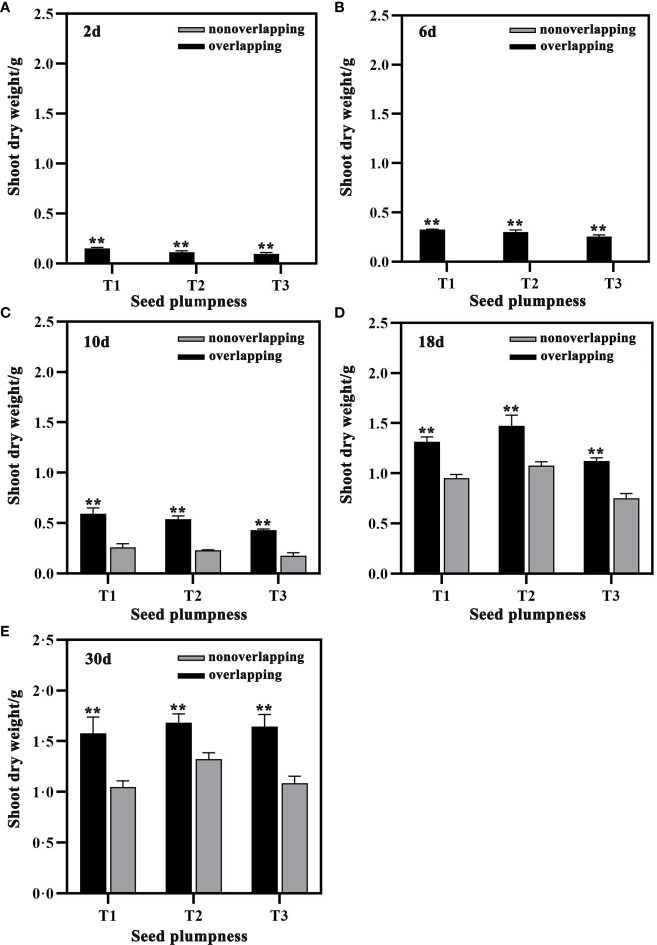
Differences in shoot dry weight of seeds with different plumpness under two seedling modes. Nonoverlapping and overlapping represent TSR and STOSE modes, respectively. 2d, 6d, 10d, 18d and 30d indicate the number of days after sowing. T1, T2 and T3 represent different types of seeds (plumped, mixed and unplumped seeds, respectively). There were three replicates for each treatment and each replicate included 100 seedlings. Bars mean SD (n=3). **, indicates that the difference in shoot dry weight of the same type of seeds under different seedling modes was highly significant (P<0.01, LSD method).

The shoot weight of seedlings under the STOSE mode was significantly higher than that under the TSR mode at all times after sowing (**Figure 6**). The shoot dry weight of the T1 and T2 treatments under the STOSE mode significantly increased at 30 days compared to that at 10 days after sowing by 166.31 and 212.02%, respectively. In contrast, T3 showed a greater increase of 281.37%, which was more significant than the previous two treatments. Moreover, the shoot dry weight of the T3 treatment under the STOSE mode increased by 144.52%, 49.11% and 51.67% at 10, 18 and 30 days after sowing, respectively, which was the highest increase among all the treatments compared to the TSR mode (**Figures 6C–E**).

**Figure 7:**
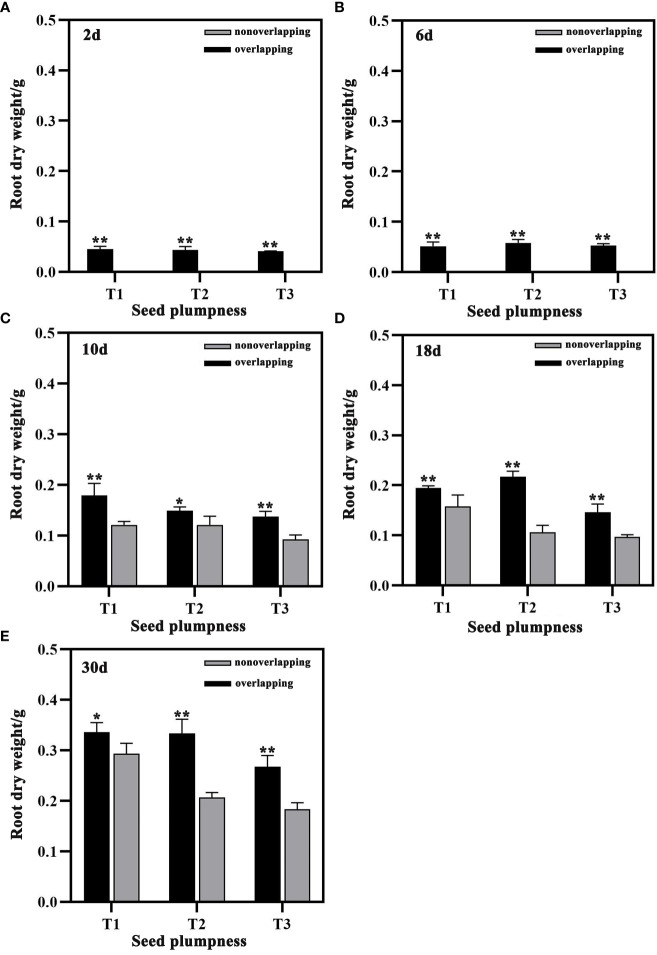
Differences in root dry weight of seeds with different plumpness under two seedling modes. Nonoverlapping and overlapping represent TSR and STOSE modes, respectively. 2d, 6d, 10d, 18d and 30d indicate the number of days after sowing. T1, T2 and T3 represent different types of seeds (plumped, mixed and unplumped seeds, respectively). There were three replicates for each treatment and each replicate included 100 seedlings. Bars mean SD (n=3). *, indicates that the difference in root dry weight of the same type of seeds under different seedling modes was significant (P<0.05, LSD method); **, indicates that the difference in root dry weight of the same type of seeds under different seedling modes was highly significant (P<0.01, LSD method).

The root dry weight of seedlings was significantly greater under the STOSE mode than the TSR mode within 30 days after sowing (**Figure 7**). Under the STOSE mode, the T3 treatment showed increases in root dry weight of 48.65% at 10 days, 49.91% at 18 days, and 46.04% at 30 days after sowing compared to the TSR mode (**Figures 7C–E**). These results indicate that the STOSE mode significantly increased the shoot and root dry weight of seedlings compared to the TSR mode. The enhancement effect was most pronounced for the unplumped seed treatment.

### 3.5 Seedling root traits

Root length, surface area, volume, and number differed significantly between the STOSE and TSR modes at all measured time points after sowing (**Table 1**). The STOSE mode improved seedling root traits compared to the TSR mode. The total root length of seedlings under the STOSE mode was significantly higher than that of seedlings under the TSR mode for all treatments (T1, T2, and T3) and at all time points after sowing. The root surface area and number were significantly higher under the STOSE mode than the TSR mode across all seed treatments, except for the T2 treatment at 30 days after sowing. Moreover, at 10, 18, and 30 days after sowing, the root volume in the T3 treatment under the STOSE mode was significantly greater than that in the TSR mode. However, the differences in root volume among the other treatments were not significant between the two modes.

**Table 1:** Difference analysis of root traits during different periods under STOSE and TSR modes.

Days after sowing/d	Seed plumpness	Root total length/(cm/plant)		Root superficial area/(cm2/plant)		Root volume/(×10-2cm3/plant)		Root number/(NO./plant)	
		nonoverlapping	overlapping	nonoverlapping	overlapping	nonoverlapping	overlapping	nonoverlapping	overlapping
2d	T1	/	2.78**	/	0.13**	/	0.25**	/	1.14**
	T2	/	2.69**	/	0.15**	/	0.29**	/	1.01**
	T3	/	2.59**	/	0.12**	/	0.20**	/	1.09**
6d	T1	/	4.56**	/	0.19**	/	0.35**	/	2.69**
	T2	/	5.18**	/	0.24**	/	0.46**	/	3.22**
	T3	/	4.97**	/	0.22**	/	0.41**	/	3.18**
10d	T1	4.95	13.49**	0.33	0.54**	0.82	1.15	3.52	8.15**
	T2	5.01	13.85**	0.33	0.56**	0.77	1.04	3.38	8.86**
	T3	4.24	15.14**	0.27	0.57**	0.62	1.08**	2.72	10.71**
18d	T1	15.48	22.94**	0.62	0.78**	1.18	1.23	7.54	14.71**
	T2	10.19	25.32**	0.42	0.86**	1.11	1.46	5.07	18.07**
	T3	13.17	20.94**	0.47	0.71**	0.85	1.15**	8.25	15.22*
30d	T1	25.21	36.38**	0.92	1.39**	1.73	2.08	16.69	30.62**
	T2	27.59	36.15**	1.02	1.18	1.76	1.77	24.37	29.23
	T3	32.22	44.78**	0.91	1.44**	1.68	2.47**	16.61	30.97**

^1)^ “/” indicates seeds not germinated. Nonoverlapping and overlapping represent TSR and STOSE modes, respectively. T1, T2 and T3 represent different types of seeds (plumped, mixed and unplumped seeds, respectively).

^2)^ ANOVA P-values and symbols are defined as: *, P<0.05; **, P<0.01.

^3)^ 2d, 6d, 10d, 18d and 30d represent days after sowing respectively.

### 3.6 N, P and K uptake

**Figure 8:**
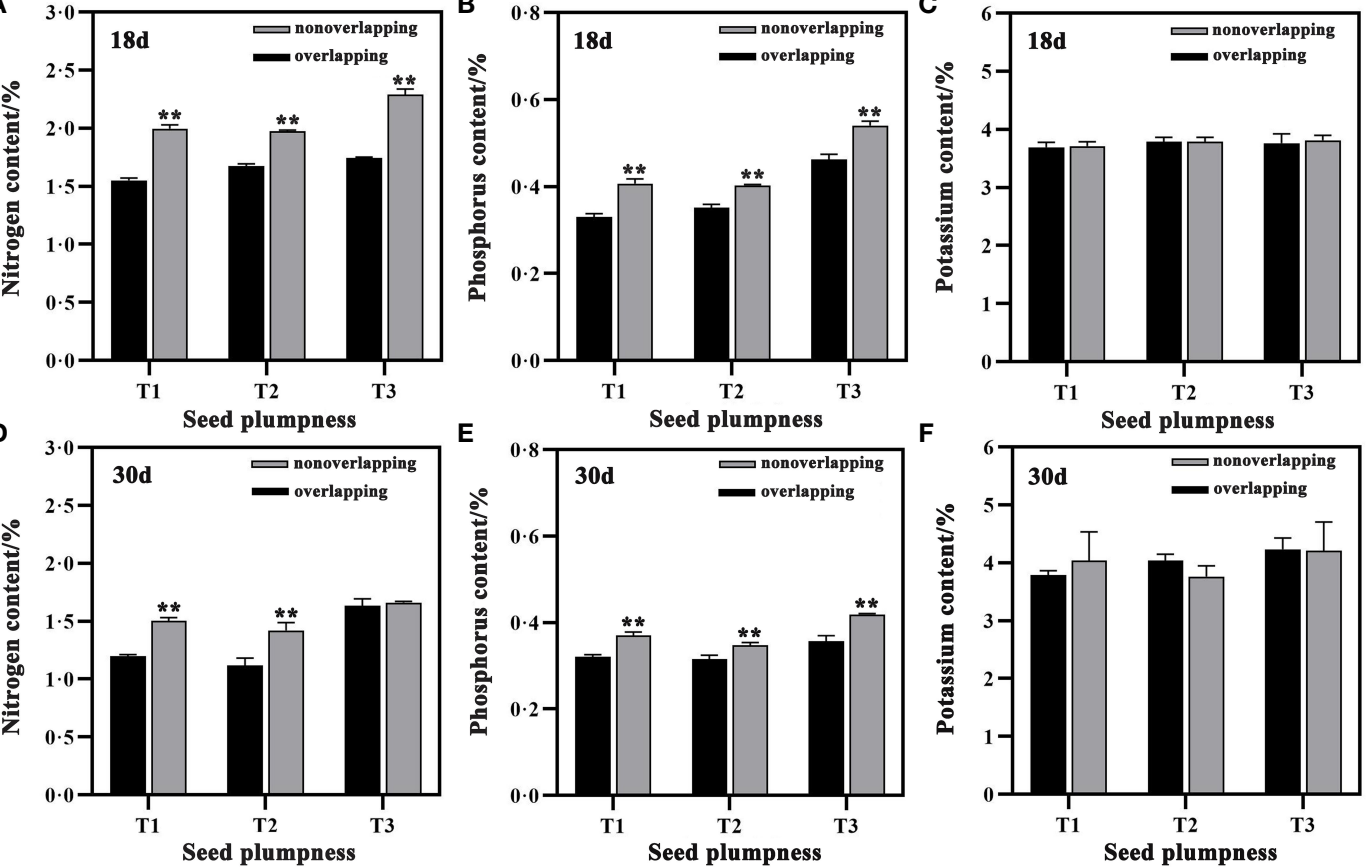
Differences in aboveground nutrient contents of seedlings between treatments under different seedling modes. Nonoverlapping and overlapping represent TSR and STOSE modes, respectively. 18d and 30d indicate the number of days after sowing. T1, T2 and T3 represent different types of seeds (plumped, mixed and unplumped seeds, respectively). Bars mean SD (n=3). **, indicates that the difference in aboveground nutrient contents of seedlings of the same type of seeds under different seedling modes was highly significant (P<0.01, LSD method).

The nitrogen (N) and phosphorus (P) contents of aboveground seedlings were significantly higher under the TSR mode than the STOSE mode at 18 and 30 days after sowing. However, the potassium (K) contents did not differ significantly between the two modes (**Figure 8**). The aboveground N and P contents of seedlings in the T3 treatment were higher than those of the other seed treatments under both the STOSE and TSR modes. The N and P contents of seedlings decreased at 30 days after sowing compared to 18 days, across all treatments. In contrast, potassium contents did not change significantly between these two time points.

**Figure 9:**
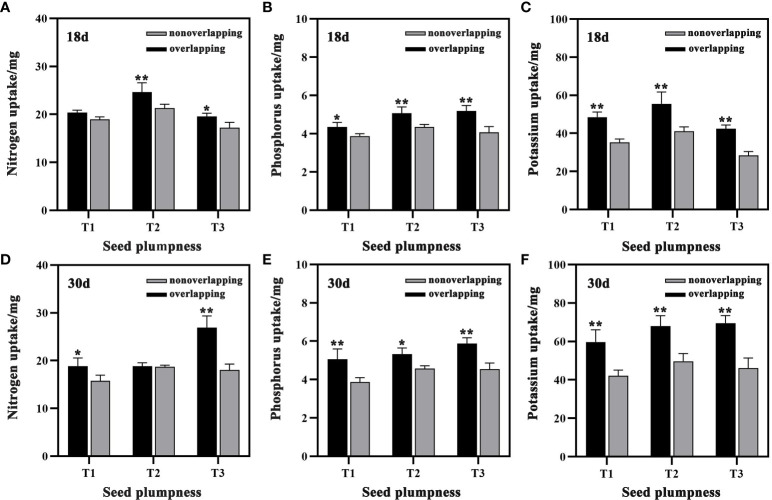
Differences in aboveground nutrient uptake of seedlings between treatments under different seedling modes. Nonoverlapping and overlapping represent TSR and STOSE modes, respectively. 18d and 30d indicate the number of days after sowing. T1, T2 and T3 represent different types of seeds (plumped, mixed and unplumped seeds, respectively). Bars mean SD (n=3). *, indicates that the difference in aboveground nutrient uptake of seedlings of the same type of seeds under different seedling modes was significant (P<0.05, LSD method); **, indicates that the difference in aboveground nutrient uptake of seedlings of the same type of seeds under different seedling modes was highly significant (P<0.01, LSD method).

The aboveground nutrient uptake of seedlings under the STOSE mode was higher than that under the TSR mode at both 18 and 30 days after sowing (**Figure 9**). Compared to the TSR mode, the aboveground N, P, and K contents of seedlings in the T3 treatment under the STOSE mode increased significantly by 13.73, 27.66 and 49.11%, respectively, at 18 days after sowing. Moreover, compared to the TSR mode, the aboveground N, P, and K contents of seedlings in the T3 treatment under the STOSE mode increased significantly by 49.31, 29.17 and 50.69%, respectively, at 30 days after sowing. These results indicate that the STOSE mode promotes greater nutrient uptake in the later stages of seedling growth compared to the TSR mode.

## 4 Discussion

### 4.1 Effects of changes in soluble sugar and starch content on seed germination under the STOSE mode

The seedling tray overlapping for seed emergence mode (STOSE mode) controls the temperature and humidity after sowing to create optimal environmental conditions for rice seed germination. This mode increases the seedling rate and helps to solve the problems of poor and uneven emergence (Zou, 2018; Chen et al., 2020). In this study, compared with the seedling tray nonoverlapping for seed emergence mode (TSR mode), the STOSE mode significantly increased the seedling rates of plumped, mixed, and unplumped seed treatments from 2 to 10 d after sowing. The STOSE mode also shortened the germination time of seeds and accelerated seedling growth and development (**Figure 2**). Furthermore, the size of the seed weight had a significant effect on seedling emergence. Generally, a higher seed weight corresponds to a higher seedling emergence rate (López-Castañeda et al., 1996). Our results also showed that plumped seeds had a significantly higher seedling emergence rate than unplumped seeds under the same seedling mode. This result agrees with previous studies. Moreover, we found that the STOSE mode greatly enhanced the seedling emergence rate of unplumped seeds compared to the TSR mode (**Figures 2D, E**). Previous studies have demonstrated that endospermic starch breakdown into soluble sugars provides necessary energy for seed germination (Wang et al., 2018). β-amylase activity plays a key role in starch activation, depolymerization, and release. Its activity can serve as an important indicator of the seedling emergence rate (Nandi et al., 1995). Under low temperature conditions, amylase activity was inhibited, resulting in significantly decreased soluble sugar content in seeds, which subsequently suppressed seed germination (Guan et al., 2009). In this study, the soluble sugar content of seeds under the STOSE mode rose sharply 2 days after sowing, while the starch content decreased significantly. Seeds of different types sprouted during this period. The soluble sugar content of seeds under the TSR mode increased greatly 6 days after sowing, coinciding with the seedling emergence period. Therefore, these results suggest that the STOSE mode promotes starch breakdown within seeds, increasing soluble sugar content and accelerating seed germination.

### 4.2 Effects of the STOSE mode on seedling growth uniformity, root traits and nutrient uptake

Seed germination rates were similar and seedling growth was uniform and robust under stabilized temperature and humidity conditions in STOSE mode (Wang et al., 2022b). Previous studies have shown that plumped seeds have higher physiological metabolism and biosynthesis capacity, which enable them to maintain higher activity and stability during drying, storage, and sowing (Gairola et al., 2011; Xie et al., 2020). Furthermore, seed activity was found to be positively correlated with seedling growth rate and uniformity (Gomes Junior et al., 2014). This means that seeds with higher plumpness exhibited faster seedling growth and uniformity. Our results demonstrated that the mixed seeds (T2) under the TSR mode had the lowest seedling growth uniformity at 18 and 30 days after sowing compared to the plumped (T1) and unplumped seeds (T3). Our hypothesis is that the difference in activity and rates of seed germination between plumped and unplumped seeds in the T2 treatment led to poorer uniformity of seedling growth. Moreover, seedling growth uniformity improved significantly in the T2 treatment under the STOSE mode compared to the TSR mode at 18 and 30 days after sowing (**Figure 5**). The results indicate that the STOSE mode contributed to an increase in the activity of the unplumped seeds in the T2 treatment, resulting in a narrower gap between their germination rates and those of the plumped seeds. This ultimately had a positive impact on the uniformity of seedling growth. The emergence of plant roots is accompanied by rapid cell division, and higher temperatures greatly facilitate this process (Teklehaimanot, 2004). Our results indicate that the average temperature was higher under the STOSE mode than the TSR mode, resulting in a significantly shorter duration for seed germination and root formation (**Table 1**).

The root system is a vital plant organ that enables water and nutrient uptake, affecting plant growth and development (Duque and Villordon, 2019; Bayala and Prieto, 2020). Previous studies have reported that crops with higher root biomass, surface area, and volume also have higher nitrogen uptake efficiency (Cassman et al., 2002; Wei et al., 2008). In this study, the total aboveground NPK uptake of seedlings in different treatments under the STOSE mode was significantly higher than that under the TSR mode at 18 and 30 days after sowing. It is speculated that the STOSE mode enhanced the development and expansion of the root system after seed germination, consequently leading to a substantial improvement in nutrient uptake efficiency by the seedlings. Previous studies have reported that the nitrogen concentration in crops decreases with increasing plant dry matter, a phenomenon known as nitrogen dilution (Cao et al., 2020). Moreover, studies on the rate of nitrogen uptake have indicated that providing sufficient nitrogen for the early stages of crop growth is the basis for obtaining higher yields (Sheehy et al., 1998). In this study, the aboveground dry matter mass of seedlings was significantly higher under the STOSE mode than the TSR mode. However, the nitrogen and phosphorus contents were lower under the STOSE mode, and this difference was highly significant. We hypothesized that this phenomenon could be attributed to two factors. First, there was a dilution of nitrogen concentration. Second, the high density of seedlings under the STOSE mode and the limited nutrient content in the substrate also played a role. Therefore, to ensure the normal growth of seedlings under the STOSE mode, it is recommended to supplement nitrogen and phosphorus nutrients during the later stages of seedling growth.

## 5 Conclusion

The STOSE mode shortened the seed germination time and accelerated seedling growth and development. Compared to the TSR mode, the STOSE mode significantly increased the seedling rate and enhanced uniformity of seedling growth. Furthermore, the STOSE mode exhibited more noticeable advantages in terms of dry matter mass, root traits, and nutrient uptake efficiency of aboveground seedlings compared to the TSR mode. Overall, the enhanced seedling rate of unplumped seeds under the STOSE mode not only reduces the number of seeds needed but also positively impacts the increase in effective number of spikes and rice yield during later stages.

## Data availability statement

The raw data supporting the conclusions of this article will be made available by the authors, without undue reservation.

## Author contributions

YG: Data curation, Investigation, Writing – original draft. MS: Investigation, Writing – original draft. YPZ: Formal analysis, Funding acquisition, Writing – review & editing. YKZ: Formal analysis, Funding acquisition, Writing – review & editing. YW: Formal Analysis, Funding acquisition, Writing – review & editing. ZW: Formal Analysis, Funding acquisition, Writing – review & editing. DZ: Conceptualization, Resources, Writing – review & editing. YBZ: Formal Analysis, Investigation, Writing – review & editing. JX: Conceptualization, Funding acquisition, Writing – review & editing. HC: Conceptualization, Funding acquisition, Writing – review & editing.
